# A longitudinal cohort study on the use of health and care services by older adults living at home with/without dementia before and during the COVID-19 pandemic: the HUNT study

**DOI:** 10.1186/s12913-024-10846-y

**Published:** 2024-04-19

**Authors:** Tanja Louise Ibsen, Bjørn Heine Strand, Sverre Bergh, Gill Livingston, Hilde Lurås, Svenn-Erik Mamelund, Richard Oude Voshaar, Anne Marie Mork Rokstad, Pernille Thingstad, Debby Gerritsen, Geir Selbæk

**Affiliations:** 1 The Norwegian National Centre for Ageing and Health (Ageing and Health), Vestfold Hospital Trust, Tønsberg, Norway; 2 Department of Geriatric Medicine, Oslo University Hospital, Oslo, Norway; 3 Department of Physical Health and Ageing, Norwegian Institute of Public Health, Oslo, Norway; 4 Research Centre for Age-Related Functional Decline and Disease (AFS), Innlandet Hospital Trust, Ottestad, Norway; 5 Division of Psychiatry, University College London, London, UK; 6 Camden and Islington NHS Foundation Trust, London, UK; 7 Health Services Research Unit, Akershus University Hospital, Oslo, Norway; 8 Institute of Clinical Medicine, University of Oslo, Oslo, Norway; 9 Centre for Research On Pandemics & Society (PANSOC), at Oslo Metropolitan University, Oslo, Norway; 10 Department of Psychiatry, University of Groningen, University Medical Center Groningen, Groningen, the Netherlands; 11 Faculty of Health Sciences and Social Care, Molde University College, Molde, Norway; 12 Department of Neuromedicine and Movement Science, Faculty of Medicine and Health Science, Norwegian University of Science and Technology, Trondheim, Norway; 13 Department of Health and Social Services, Trondheim Municipality, Trondheim, Norway; 14 Department of Primary and Community Care, Research Institute for Medical Innovation, Radboudumc Alzheimer Center, Radboud University Medical Center, Nijmegen, Netherlands; 15 Department of Geriatric Medicine, Oslo University Hospital, Oslo, Norway; 16 Institute of Clinical Medicine, Faculty of Medicine, University of Oslo, Oslo, Norway

**Keywords:** COVID-19, Dementia, Health care services, Older adults, Longitudinal cohort study

## Abstract

**Background:**

Older adults and people with dementia were anticipated to be particularly unable to use health and care services during the lockdown period following the COVID-19 pandemic. To better prepare for future pandemics, we aimed to investigate whether the use of health and care services changed during the pandemic and whether those at older ages and/or dementia experienced a higher degree of change than that observed by their counterparts.

**Methods:**

Data from the Norwegian Trøndelag Health Study (HUNT4 70 + , 2017–2019) were linked to two national health registries that have individual-level data on the use of primary and specialist health and care services. A multilevel mixed-effects linear regression model was used to calculate changes in the use of services from 18 months before the lockdown, (12 March 2020) to 18 months after the lockdown.

**Results:**

The study sample included 10,607 participants, 54% were women and 11% had dementia. The mean age was 76 years (SD: 5.7, range: 68–102 years). A decrease in primary health and care service use, except for contact with general practitioners (GPs), was observed during the lockdown period for people with dementia (*p* < 0.001) and those aged ≥ 80 years without dementia (*p* = 0.006), compared to the 6-month period before the lockdown. The use of specialist health services decreased during the lockdown period for all groups (*p* ≤ 0.011), except for those aged < 80 years with dementia. Service use reached levels comparable to pre-pandemic data within one year after the lockdown.

**Conclusion:**

Older adults experienced an immediate reduction in the use of health and care services, other than GP contacts, during the first wave of the COVID-19 pandemic. Within primary care services, people with dementia demonstrated a more pronounced reduction than that observed in people without dementia; otherwise, the variations related to age and dementia status were small. Both groups returned to services levels similar to those during the pre-pandemic period within one year after the lockdown. The increase in GP contacts may indicate a need to reallocate resources to primary health services during future pandemics.

**Trial registration:**

The study is registered at ClinicalTrials.gov, with the identification number NCT 04792086.

## Background

In Norway, similar to most European countries [1–3], the first wave of the COVID-19 pandemic lasted from 12 March to 15 June 2020 [4]. During this period, strict infection control measures were introduced to minimise the number of infected people. Health and care services were reduced or locked down, because health professionals were transferred to COVID-19-related services, or hospital wards were reserved for COVID-19 patients. Facilities such as day care services were closed to prevent the spread of infection through social contact, and some services were employed with digital technology. People were urged to stay at home to maintain social distancing and prevent the spread of the virus [4].

The strict infection control measures aimed mainly to prevent people from hospitalisation and/or death by COVID-19. By 13 November 2022 (last published data), Norway recorded 4,399 cumulative COVID-19-related deaths, of which approximately two-thirds occurred in 2022 (in people of an average age of 85.6 years in 2022) [5]. From March 2020 to March 2021, compared to the mean all-cause mortality from 2016 to 2019 as a reference, Norway recorded significantly lower all-cause mortality than those recorded by other European Union countries [6], indicating that Norway had a successful public health strategy. The topic being raised in the present paper, is how infection control measures affected the use of health and care services by the older population, to better prepare ourselves for future health crisis like a pandemic.

Older adults are particularly vulnerable to COVID-19 and at a higher risk of hospitalisation and death [7]. People with dementia are anticipated to have an even higher risk of mortality than that of people without dementia, because of an impaired immune system [8]. Fearing the virus, some older adults personally imposed strict infection control measures and cancelled scheduled healthcare appointments. A German study, including participants aged ≥ 73 years, has reported that approximately 30% of the participants reduced or cancelled their medical consultations during the first wave of the pandemic [1]. A qualitative study including participants aged 65–79 years from Portugal, Brazil, and the United Kingdom has reported that the majority refrained from face-to-face contact with their family doctors in the first wave of the pandemic, as it implied using public transport making social distancing difficult [2]. Some health and care services have been replaced with online or telephone consultations, which have been beneficial for some parts of the population and challenging for others, especially older adults [2, 3, 9].

People with dementia often need health and care services and practical assistance in their homes to manage their everyday lives [10]. A Norwegian study including 105 caregivers of people with dementia has reported that 60% experienced a reduction or full cessation of formal care during the first wave of the pandemic as the services were cancelled by the service provider [11]. This is in line with studies from Sweden and the USA, which reported a significant drop in the use of health and care services during this period [12, 13]. However, how the use of primary and specialist healthcare services affected older adults, including people with dementia, as society began a cautious reopening after the first wave of the pandemic remains unclear. A study from the USA conducted a predictive analysis for the post-lockdown period (June 2020–October 2021) on inpatient, outpatient, and emergency services. They found that people with mild cognitive impairment (MCI), Alzheimer’s disease, and related dementia experienced greater and more sustained disruptions in primary and specialist health and care service use than those experienced by people without MCI or dementia [13].

In the present study, we used a large population-based dataset from the Norwegian Trøndelag Health Study (HUNT) [14], linked to national registry data on primary and specialist health and care services, to investigate whether the use of health and care services changed during the pandemic, and those with older ages and/or dementia had a higher degree of change than that observed in their counterparts.

## Methods

### Study design and setting

**Fig. 1:**
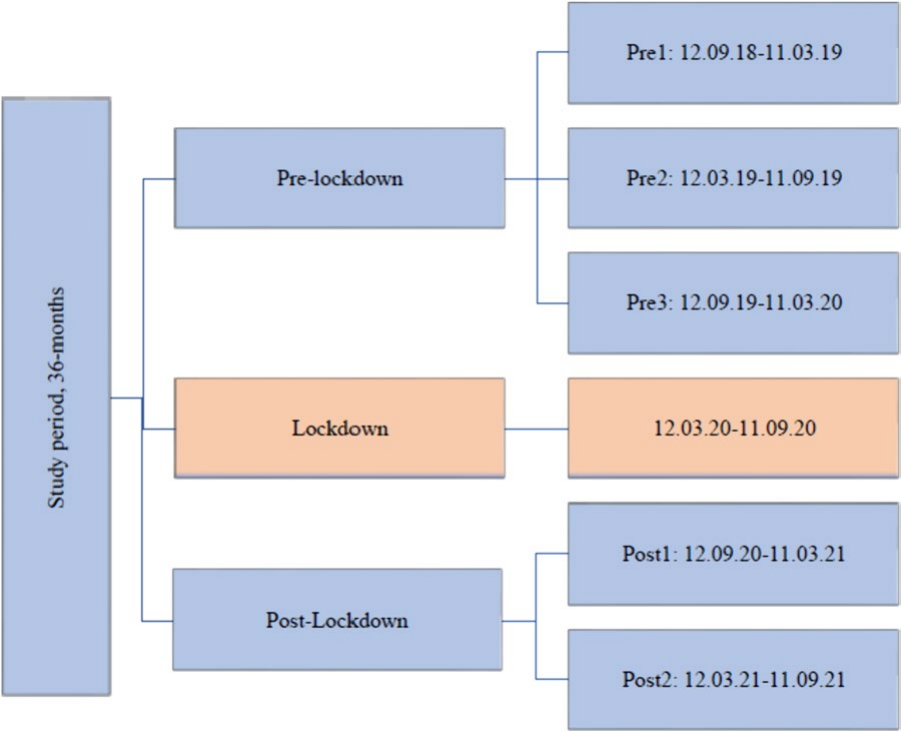
Flow-chart of the study periods

We used a longitudinal cohort design, linking participant data on sex, year of birth, and cognitive status from the HUNT4 70 + survey with later registry data on the use of health and care services from 12 September 2018 to 11 September 2021. This time period equals 18 months before- and 18 months after the Norwegian lockdown on 12 March 2020. This 36-month period was grouped into six periods of six months each, including three pre-lockdown periods (pre1, pre2, and pre3), one lockdown period, and two post-lockdown periods (post1 and post2) (Fig. 1). We included a longer lockdown period than the generally denoted period from March to June 2020, as the reopening started slowly, and many older adults imposed strict social distancing on themselves. The next period, 12 September 2020 to 11 March 2021 also included periods with restrictions on social life and activity, such as a maximum of five people gathering and recommendations for wearing a face mask where maintaining distance is difficult. In the last period from 12 March to 11 September 2021, all infection control measures were gradually lifted until Norway was completely reopened on 25 September 2021 [4]. Trøndelag, the county where the study was conducted, followed national infection control regulations.

### Participants

**Fig. 2:**
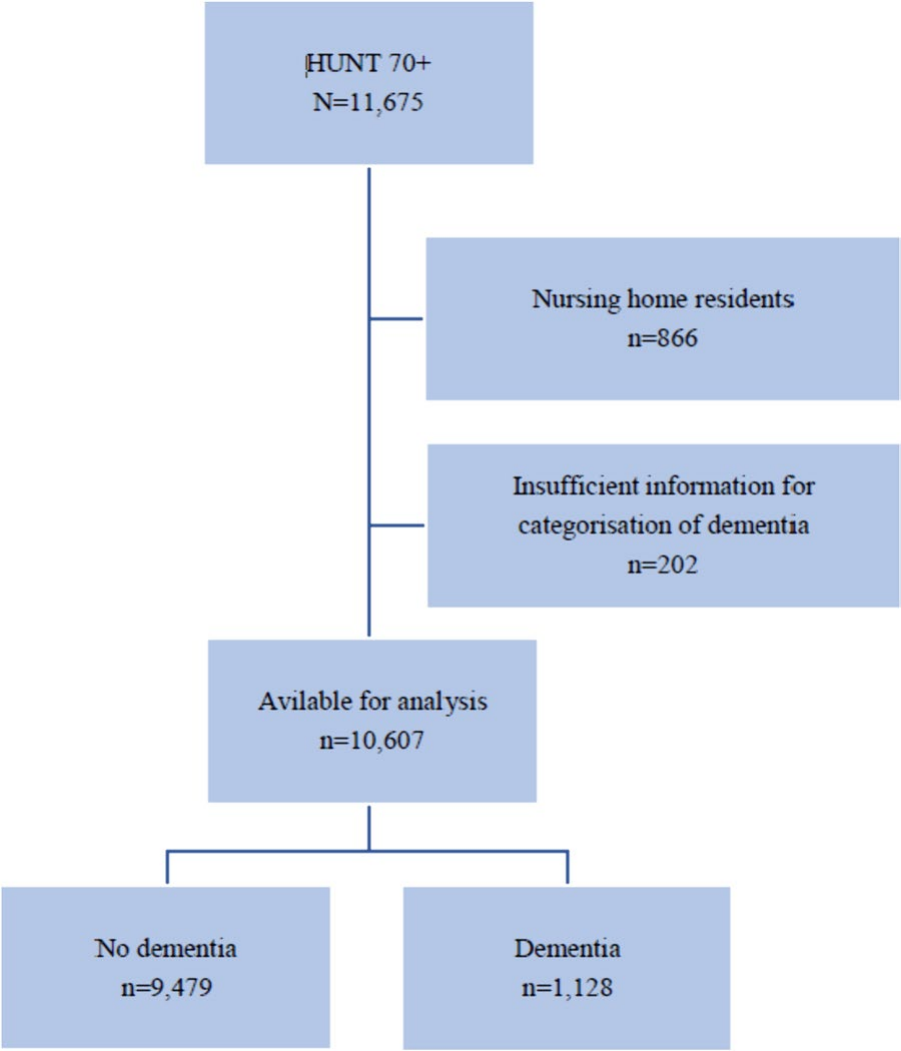
Flow-chart of included participants. HUNT4 70 + : The fourth wave of the Trøndelag health study, 70 year and older cohort

The study included participants aged > 70 years in the fourth wave of the HUNT Study (HUNT4 70 +), which took place between September 2017 and March 2019. The HUNT is a population-based study that has invited the entire adult population from the same geographic area, North-Trøndelag, in four waves, first time in 1984 [14]. As North-Trøndelag does not comprise any large cities, a random sample of people aged > 70 years from a city in Trondheim (212,000 inhabitants) was also invited. In total, 11,675 participants were included, with 9,930 from North-Trøndelag (response rate 51%) and 1,745 from Trondheim (response rate 34%). We do not judge that there is likely to be any systematic bias introduced by the difference in response rates in different municipalities as the people living at home are similar populations.”. The participants answered a questionnaire that included socio-demographic and clinical data, and they attended a comprehensive clinical evaluation by health professionals [15]. Participants without sufficient information on their cognitive status (*n* = 202) and nursing home residents (*n* = 866) were excluded (Fig. 2). The mean age (76 years, SD 5.7 years) of those included was lower than that of those excluded (82 years, SD 7.9) (*p* < 0.001). The study population included 10,607 participants with complete data on cognitive status. We do not have information on dementia status on the population not included in HUNT4 70 + .

### Dementia diagnosis

Two specialists from a diagnostic workgroup of nine medical doctors with comprehensive scientific and clinical expertise (geriatrics, old-age psychiatry, or neurology) independently diagnosed each patient with dementia using the Diagnostic and Statistical Manual of Mental Disorders-5 [16]. Discrepancies were resolved and consensuses were obtained by the involvement of a third expert. During the diagnostic process, the experts had access to all relevant information from the HUNT4 70 + dataset, such as education, function in activities of daily living, neuropsychiatric symptoms, onset and course of cognitive symptoms, cognitive tests (the Montreal Cognitive Assessment (MoCA) scale [17] and the Word List Memory Task (WLMT) [18], and structured interviews with the closest family proxy. A more comprehensive description of the diagnostic process has been published [15].

### Health and care services

Data from two national registries were collected for the entire study period, from September 2018 to September 2021. Health and care services used in primary health care were obtained from The Norwegian Registry of Primary Health Care [19]. This registry includes individual-level data on municipal health services (contacts with general practitioners (GPs), emergency rooms, and physiotherapists) and care services (care, such as home nurses, and practical assistance in the recipient’s home, day care, respite services and short-term nursing home stays, municipal housing, and nursing home admission) [20]. Information on the use of specialist health services was based on data from the Norwegian Patient Registry (NPR) [21]. The NPR holds individual-level data on patients’ use of specialist health services (contacts with somatic hospitals, mental health care, and rehabilitation institutions). The NPR also registers whether the contact was an outpatient consultation, hospitalisation, or day-treatment [20].

### Analysis

Data were analysed using the STATA 16 software [22]. Participant characteristics are reported as means with SD, frequencies, or percentages, as appropriate. Those who were admitted to a nursing home (*n* = 364) or died (*n* = 821) during the study period were censored and participated in only half of the person-time during the study period. Duplicates were removed (3,293 observations). The mean number of health and care services per person in each period (with 95% confidence interval [CI]) was predicted from a multilevel mixed-effects linear regression model with random intercept, where random effects varied across the individuals. In the regression model, the number of services per person was the outcome variable and sex, age, cognitive status (no dementia/dementia), and period were covariates.Age and cognitive status are relevant confounders to address the aim of the present study, and sex is included as a key sociodemographic measure in epidemiological research. [23, 24]. To allow for different time trends by cognitive status group, the interaction term period by cognitive status was included in the regression model. In the predictions, the adjusted variables were fixed at their mean values. The significance level was set at *p* < 0.05. To investigate the use of health and care services before and during the pandemic, the number of care services implemented within each period and the number of contacts within each period for primary and specialist health services were aggregated. Hence, for care services, we used the date on which the service was implemented, for example the date on which practical assistance at home was implemented. For health services, we used the date when the service occurred, for example, the date a person had contact with a GP or the date a person had contact with a hospital, either for outpatient consultation, hospitalisation, or day-treatment.

In the Results section, we report significant differences between the lockdown period and all the pre- and post-lockdown periods, and between pre2 and post2, as these periods comprise the same seasonal months, one year before and one year after the lockdown, respectively.

## Results

The study included 10,607 participants, of whom 54% were women, and 11% had dementia (Table 1). The mean age of the participants on 1 January 2017 was 76 years (SD 5.7, range: 68–102 years), and 7,769 participants (73%) were < 80 years old. During the 36-month follow-up period, the study sample was reduced by 10% (from 10,607 to 9,568) due to censoring for death and/or nursing home admission (Table 2). The dropout rate was higher in those with dementia than in those without dementia (37% vs. 7%, *p *< 0.001). During these 36-months, the total number of contacts with primary health services was 554,061, which corresponded to 9.2 contacts per person per 6-month period (Table 3). People with dementia had more contact with health services in the municipality than the contact made by those without dementia (11.3 vs. 8.8 contacts per person per 6-month period, *p* < 0.001). The total number of care services implemented for our study population was 20,411, which corresponded to 0.3 care services per person per 6-month period. People with dementia received more care services than those received by people without dementia (1.2 vs. 0.2 care services per person per 6-month period, *p* < 0.001). The total number of contacts with specialist health services was 141,994, which corresponded to 2.3 contacts per person per 6-month period. People with dementia had less contact with specialist health services than the contact made by those without dementia (2.2 vs. 2.6 contacts per person per 6-month period, *p* < 0.001).

**Table 1:** Description of the study sample, across dementia status

Study sample	Total	No dementia	Dementia
Participants (%)	10,607	9,479 (89)	1,128 (11)
Women (%)	5,705 (54)	5,077 (89)	628 (11)
Age (mean, SD)	76.4 (5.9)	75.9 (5.5)	81.2 (7.1)

**Table 2:** Number of participants reported from baseline to end of study, across dementia status, including participants censored due to nursing home admission or death

		**All**			**No dementia**			**Dementia**		
Periods	Dates	Number of persons at start of each period	Number of persons censored per period	Period Person Time*	Number of persons at start of each period	Number of persons censored per period	Period Person Time*	Number of persons at start of each period	Number of persons censored per period	Period Person Time*
Pre-lockdown 1	12.09.18–11.03.19	10,607	131	10,541.5	9,479	69	8,444.5	1,128	62	1,097.0
Pre-lockdown 2	12.03.19–11.09.19	10,476	174	10,389.0	9,410	95	9,362.5	1,066	79	1,026.5
Pre-lockdown 3	12.09.19–11.03.20	10,302	190	10,207.0	9,315	107	9,261.5	987	83	945.5
**Lockdown**	12.03.20–11.09.20	10,112	180	10,022.0	9,208	114	9,151.0	904	66	871.0
Post-lockdown 1	12.09.20–11.03.21	9,932	171	9,846.5	9,094	104	9,042.0	838	67	804.5
Post-lockdown 2	12.03.21–11.09.21	9,761	193	9,664.5	8,990	136	8,922.0	771	57	742.5

*Persons censored add 0.5 period person time, while those not censored adds 1 person time per period

**Table 3:** Number of registrations within primary health and care services and specialist health services during the pre-lockdown, lockdown and post-lockdown periods, across age groups and dementia status

	Number of services, n (mean*)						
**Registry**	**Total**	**No dementia**			**Dementia**		
	*n* = 10,607	** < 80 years** *n* = 7282	≥ **80 years** *n* = 2197	**Total** *n* = 9,479	** < 80 years** *n* = 487	≥ **80 years** *n* = 641	**Total** *n* = 1128
Primary health services (mean*)	554,061 (9.2)	362,525 (8.5)	129,696 (10.6)	492,221 (8.8)	27,916 (10.7)	33,924 (11.7)	61,840 (11.3)
Pre-lockdown 1	96,152 (9.1)	60,946 (8.4)	23,034 (10.6)	83,980 (8.9)	4,961 (10.2)	7,256 (11.8)	12,172 (11.1)
Pre-lockdown 2	91,085 (8.8)	57,509 (8.0)	21,982 (10.3)	79,491 (8.5)	4,938 (10.6)	6,656 (11.8)	11,594 (11.3)
Pre-lockdown 3	97,578 (9.6)	63,937 (8.9)	22,807 (11.0)	86,744 (9.4)	4,712 (10.6)	6,122 (12.2)	10,834 (11.5)
**Lockdown**	81,578 (8.1)	52,850 (7.4)	19,555 (9.7)	71,405 (7.9)	4,180 (9.8)	4,993 (11.2)	9,173 (10.5)
Post-lockdown 1	101,088 (10.3)	67,446 (9.5)	23,496 (12.0)	90,942 (10.1)	4,986 (12.2)	5,160 (13.0)	10,146 (12.6)
Post-lockdown 2	86,580 (9.0)	59,837 (8.5)	18,822 (10.0)	78,659 (8.1)	4,184 (10.7)	3,737 (10.6)	7,921 (10.7)
Primary care services (mean*)	20,411 (0.3)	5,083 (0.1)	8,563 (0.7)	13,646 (0.2)	2,204 (0.9)	4,561 (1.6)	6,765 (1.2)
Pre-lockdown 1	3,675 (0.3)	711 (0.1)	1,353 (0.6)	2,064 (0.2)	431 (0.9)	1,180 (2.0)	1,611 (1.5)
Pre-lockdown 2	3,329 (0.3)	704 (0.1)	1,304 (0.6)	2,008 (0.2)	381 (0.8)	940 (1.7)	1,321 (1.3)
Pre-lockdown 3	4,324 (0.4)	982 (0.1)	1,856 (0.9)	2,838 (0.3)	481 (1.1)	1,005 (2.0)	1,486 (1.6)
**Lockdown**	2,954 (0.3)	814 (0.1)	1,344 (0.7)	2,158 (0.2)	282 (0.7)	514 (1.2)	796 (0.9)
Post-lockdown 1	3,444 (0.3)	995 (0.1)	1,544 (0.8)	2,539 (0.3)	391 (1.0)	514 (1.3)	905 (1.1)
Post-lockdown 2	2,685 (0.3)	877 (0.1)	1,162 (0.6)	2,039 (0.2)	238 (0.6)	408 (1.2)	646 (0.9)
Specialist health services (mean*)	141,994 (2.3)	98,020 (2.3)	32,102 (2.6)	130,122 (2.3)	6,647 (2.6)	5,225 (1.8)	11,872 (2.2)
Pre-lockdown 1	24,485 (2.3)	16,272 (2.2)	5,749 (2.6)	22,021 (2.3)	1,293 (2.7)	1,171 (1.9)	2,464 (2.2)
Pre-lockdown 2	23,236 (2.2)	15,543 (2.2)	5,507 (2.6)	21,050 (2.2)	1,060 (2.3)	1,126 (2.0)	2,186 (2.1)
Pre-lockdown 3	25,701 (2.5)	17,661 (2.5)	5,938 (2.9)	23,599 (2.5)	1,119 (2.5)	983 (2.0)	2,102 (2.2)
**Lockdown**	20,784 (2.1)	14,557 (2.0)	4,638 (2.3)	19,195 (2.1)	979 (2.3)	610 (1.4)	1,589 (1.8)
Post-lockdown 1	24,796 (2.5)	17,538 (2.5)	5,352 (2.7)	22,890 (2.5)	1,180 (2.9)	726 (1.8)	1,906 (2.4)
Post-lockdown 2	22,992 (2.4)	16,449 (2.3)	4,918 (2.6)	21,367 (2.4)	1,016 (2.6)	609 (1.7)	1,625 (2.2)

*Mean number of services per person per period

Primary health services: contacts with general practitioners, emergency rooms and physiotherapists.

Primary care services: number of services implemented of care and practical assistance in the home, day care, respite services and short-term institutional stays, municipal housing, and nursing home admission.

Specialist health services: contacts with somatic hospitals, mental health care, and rehabilitation institutions.

Pre-lockdown 1 = 12.09.18–11.03.19, Pre-lockdown 2 = 12.03.19–11.09.19, Pre-lockdown 3 = 12.09.19–11.03.20, Lockdown = 12.03.20–11.09.20, Post-lockdown 1 = 12.09.20–11.03.21, Post-lockdown 2 = 12.03.21–11.09.2.

### Primary health and care services

#### Health services

During the 36-month study period, contact with GPs was the most used health service (66%), followed by physiotherapy services (30%), and contact with GPs in emergency rooms (4%).

The following model only presents contact with GPs, including GPs in emergency rooms, as contact with GPs was the most frequently used primary health service.

**Fig. 3:**
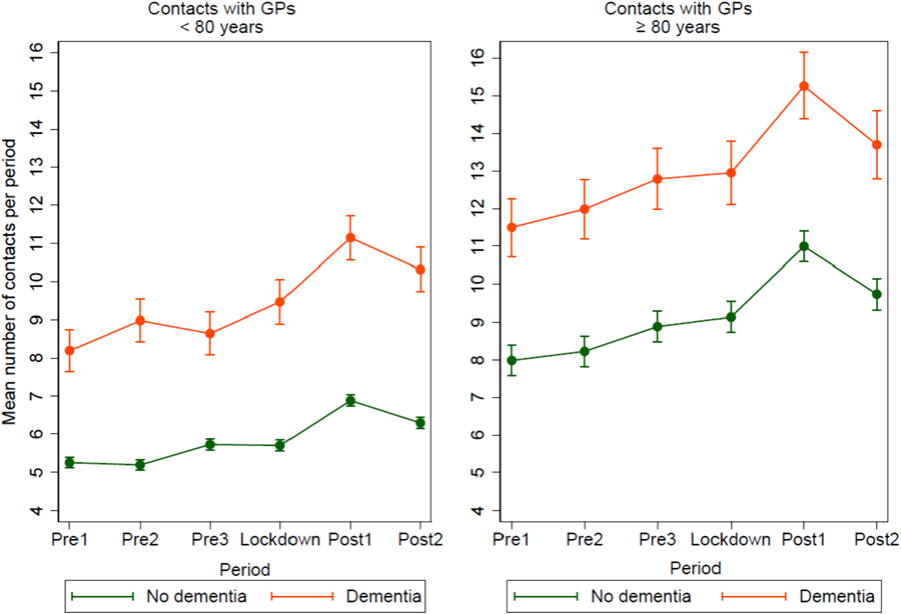
Mean number of registered contacts with general practitioners (GPs) per period, pre-lockdown, during lockdown and post-lockdown, including GPs at emergency rooms, for participants < 80 versus ≥ 80 years, divided in people with- or without dementia. Mean number of contacts was predicted in a mixed-effects linear regression model adjusted by period, cognitive status, sex, age, and the interaction period*cognitive status. In the predictions, the adjustment variables age and sex were fixed at the mean values

The age- and sex-adjusted model (Fig. 3) shows that for those aged < 80 years with dementia, the mean number of GP contacts during the lockdown period was higher than that during pre1 (1.27, *p* < 0.001) and pre3 (0.82, *p* = 0.002) and lower than that during post1 (1.67, *p* < 0.001) and post2 (0.84, *p* < 0.002). The mean number of GP contacts during post2 was higher than that during pre2 (0.32, *p* < 0.001).

For those without dementia, the mean number of GP contacts during the lockdown was higher than that during pre1 (0.45, *p* < 0.001) and pre2 (0.51, *p* < 0.001) and lower than that during post1 (1.18, *p* < 0.001) and post2 (0.59, *p* < 0.001). The mean number of GP contacts during post2 was higher than that during pre2 (1.11, *p* < 0.001).

For those aged ≥ 80 years with dementia, the mean number of GP contacts during the lockdown was higher than that during pre1 (1.45, *p* < 0.001) and pre2 (0.96, *p* = 0.015) and lower than that during post1 (2.31, *p* < 0.001). The mean number of GP contacts during post2 was higher than that during pre2 (1.72, *p* < 0.001).

For those without dementia, the mean number of GP contacts during the lockdown was higher than that during pre1 (1.15, *p* < 0.001) and pre2 (0.91, *p* < 0.001) and lower than that during post1 (1.86, *p* < 0.001) and post2 (0.60, *p* < 0.002). The mean number of GP contacts during post2 was higher than that during pre2 (1.51, *p* < 0.001).

#### Care services

During the 36-month study period, care and practical assistance at home represented the largest service group (69%), followed by short-term nursing home stays and respite services (21%), nursing home admissions (4%), municipal housing (3%), and day care services (4%). The following model presents all combined care services.

**Fig. 4:**
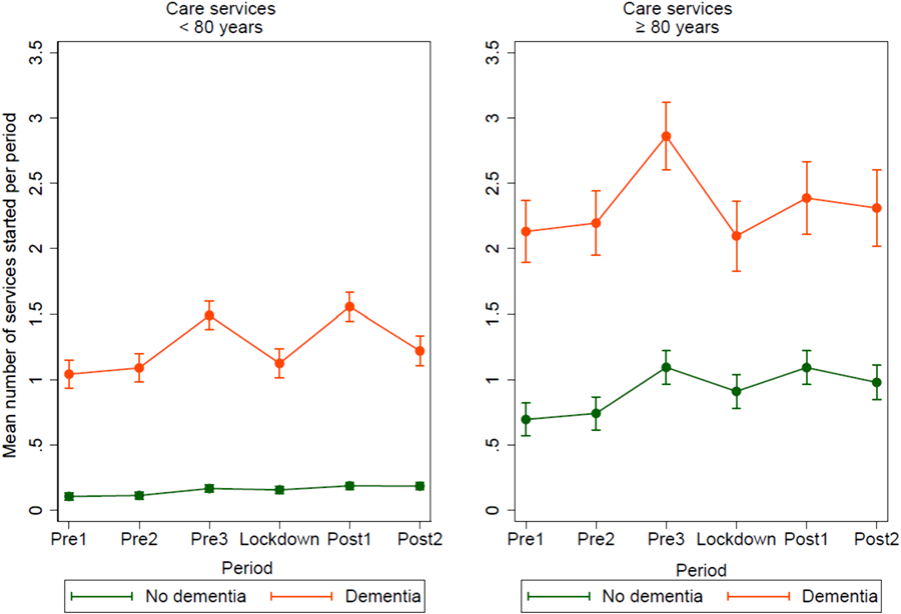
Mean number of care services implemented per period, pre-lockdown, during lockdown and post-lockdown, as health care and practical assistance in the home, day- and respite services, short-term institutional stay, and nursing home admission, for participants < 80 versus ≥ 80 years, divided in people with- and without dementia. Mean number of care services implemented was predicted in a mixed-effects linear regression model adjusted by period, cognitive status, sex, age, and the interaction period*cognitive status. In the predictions, the adjustment variables age and sex were fixed at the mean values

The age- and sex-adjusted model (Fig. 4) shows that for those aged < 80 years with dementia, the mean number of care services implemented during the lockdown was lower than that during pre3 (0.37, *p* < 0.001) and post1 (0.43, *p* < 0.001). The mean number of care services implemented in post2 was higher than that during pre2 (0.13, *p* = 0.039).

For those without dementia, the mean number of care services implemented during the lockdown was higher than that during pre1 (0.5, *p* = 0.001) and pre2 (0.04, *p* = 0.005) and lower than that during post1 (0.03, *p* = 0.044). The mean number of care services implemented during post2 was higher than that during pre2 (0.07, *p* < 0.001).

For those aged ≥ 80 years with dementia, the mean number of care services implemented during the lockdown was lower than that during pre3 (0.76, *p* < 0.001).

For those without dementia, the mean number of care services implemented during the lockdown was higher than that during pre1 (0.22, *p* = 0.001) and pre2 (0.17, *p* = 0.011) and lower than that during pre3 (0.18, *p* = 0.006) and post1 (0.18, *p* = 0.007). The mean number of care services implemented during post2 was higher than that during pre2 (0.24, *p* < 0.001).

### Specialist health services

During the study period, service provision from somatic hospitals was the most used service (96%), followed by mental health care (3%), and treatment at a rehabilitation institution (1%). Somatic hospital services included outpatient consultations (88%), hospitalisation (9%), and daily treatment (3%). The following model only presents contacts with somatic hospital services, as this is the most frequently used specialist health service.

**Fig. 5:**
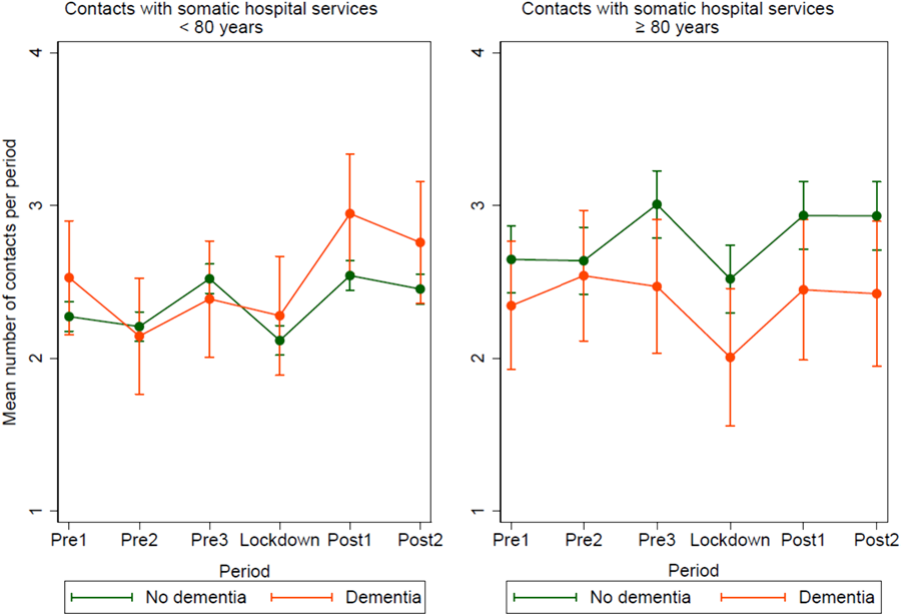
Mean number of registered contacts with somatic hospital services per period, pre-lockdown, during lockdown and post-lockdown, for participants < 80 versus ≥ 80 years, divided in people with- or without dementia. Mean number of contacts was predicted in a mixed-effects linear regression model adjusted by period, cognitive status, sex, age, and the interaction period*cognitive status. In the predictions, the adjustment variables age and sex were fixed at the mean values

The age- and sex-adjusted models (Fig. 5) show that for those aged < 80 years with dementia, the mean number of contacts with somatic hospital services during the lockdown was lower than that during post1 (0.67, *p* = 0.002) and post2 (0.48, *p* = 0.025). The mean number of contacts with somatic hospital services in post2 was higher than that during pre2 (0.61, *p* = 0.004).

For those without dementia, the mean number of contacts with somatic hospital services during the lockdown was lower than that during pre1 (0.16, *p* = 0.002), pre3 (0.40, *p* < 0.001), post1 (0.43, *p* < 0.001), and post2 (0.34, *p* < 0.001). The mean number of contacts with somatic hospital services in post2 was higher than that during pre2 (0.25, *p* < 0.001).

For those aged ≥ 80 years with dementia, the mean number of contacts with somatic hospital services during the lockdown was lower than that during pre2 (0.54, *p* = 0.003), pre3 (0.46, *p* = 0.011), post1 (0.44, *p* = 0.022), and post2 (0.42, *p* = 0.040).

For those without dementia, the mean number of contacts with somatic hospital services during the lockdown was lower than that during pre3 (0.49, *p* < 0.001), post1 (0.41, *p* < 0.001), and post2 (0.41, *p* < 0.001). The mean number of contacts with somatic hospital services in post2 was higher than that during pre2 (0.29, *p* = 0.001).

## Discussion

This population-based study revealed that people with dementia experienced a larger decrease in the use of primary care services implemented during the lockdown than that experienced by people without dementia. Contact with GPs was maintained at a normal level or increased in both groups during the lockdown. The use of specialist health services decreased in both groups during the lockdown period except for those aged < 80 years with dementia. The use of primary health and care services, and specialist health services was at the same or higher-level post-lockdown (post2) as pre-lockdown (pre2). Collectively, these results indicate an increased burden on primary health services during the lockdown.

### Primary health and care services

#### Health services

Both cognitive groups had a similar number of GP contacts during lockdown as pre-lockdown. Those aged < 80 years with dementia experienced an increased number of GP contacts during the lockdown compared to the numbers during the 6-month period before the lockdown (pre3). Furthermore, all the groups had an increased number of GP contacts in the first 6-months period post-lockdown (post1). Unfortunately, we were unable to identify whether the consultations were digital in our material; however, digital consultations may have contributed to maintaining contact with GPs during the pandemic. This corresponds with the results of a previous study which has reported that the Norwegian population experienced an increased use of telephone and video consultations during the pandemic [3]. However, a survey during the pandemic in the same study population as that of the present study (HUNT4 70 +) revealed that only 8% reported contact with healthcare professionals via screen-based media or telephone at least once a month during the pandemic [9]. In addition, a survey of video consultations among Norwegian GPs during the pandemic revealed that video consultations were unsuitable for the oldest population [25].

The results of the present study may indicate that GPs managed to serve older adults in Norway during the pandemic and that the cancellations of medical consultations described among older adults in other countries [1, 2] have been less extensive in Norway. Meanwhile, contact with GPs may have shifted towards more severe cases, where patients in need of specialist health services who postponed contact because of COVID-19 used the primary care service. In addition, the increase in GP contact post-lockdown may imply an increased stress level among older adults and an increase in health problems during the lockdown, which will be discussed in more detail in a later section.

#### Care services

Our finding that people with dementia experienced a larger decrease in the number of care services implemented during the lockdown than that experienced by people without dementia is in line with those of earlier studies [11, 13]. This is most likely a consequence of the fact that people with dementia use care services more often and thus, are more affected when such services are reduced or locked down. Interestingly, those with dementia in both age groups experienced a significant increase in new services implemented in the 6-month period before the lockdown (pre3). However, the possible cause for the increase in care services implemented, such as a reduction in other services or societal changes during this period, remains unconfirmed. The most likely explanation is an increase in service needs related to dementia progression, although some random fluctuations cannot be ruled out.

Care service providers have reported a deterioration in older adults’ health during the pandemic related to the absence of social support, which, in turn, has led to less support with meals, practical help, and physical activity [26]. Next of kin reported that people with dementia had a reduction in cognitive- and functional abilities because of the limited possibility of meaningful activities and mental stimulation when they had to stay at home [27, 28]. Furthermore, a lack of social connections [29] and perceived social support [30] are associated with cognitive decline and depression. Based on these findings, it can be assumed that the need for care services may be the same or higher post-lockdown than that in the 6-month period before the pandemic (pre3). However, the number of care services implemented post-lockdown (post2) was at the same level as that at pre-lockdown (pre2).

#### Specialist health services

This study revealed that somatic hospital services for those aged ≥ 80 years were the only services with a lower level of contact during the lockdown period than during the comparable pre-lockdown period (pre2). Both those with and without dementia had a decrease in somatic hospital services during the lockdown period, compared to the 6-months period before the lockdown. This corresponds with findings from an Italian study conducted in the autumn of 2020, reporting that hospitalisations and outpatient visits among older adults aged ≥ 65 years were reduced by 18.3% during the pandemic [31].

The decrease in the use of somatic hospital services during the lockdown observed in the present study was most likely related to strict infection control measures that prevented a widespread COVID-19 outbreak. Furthermore, it may be interpreted as a precautionary measure taken to minimize the risk of exposing older adults to hospitals, where a considerable number were affected by COVID-19. Hospital services experienced the greatest decline in activity during the lockdown due to preparedness for COVID-19 patients [32]. In the present study, all the groups returned to the same or a higher level of contact with somatic hospital services post-lockdown (post2), than they had pre-lockdown (pre2). Conversely, a study from the USA has suggested that people with dementia or MCI would experience more sustained disruption in primary and specialist health services than that experienced by people without such diagnoses [13]. Another study from the USA has revealed that those with comorbidities, often present among people with dementia, were at a higher risk of delayed or missed care during the pandemic [33]. The contrast in the findings may be related to differences in the healthcare system. In addition, the World Health Organization has reported disruptions in both primary and specialist health services worldwide two years into the pandemic. High-income countries reported fewer service disruptions than those reported by low-income ones [34]. The increase in GP contact post-lockdown in the present study may indicate that primary health services have been able to relieve specialist health services in Norway, so that people with dementia and others in need of specialist health services may be prioritised.

The variation in the frequency of contact with both somatic hospital services and GPs may be observed in the context of normal seasonal variations, where contact might be higher in the autumn and winter months (pre1, pre3, and post2) than in the spring and summer months (pre2, lockdown, and post2). However, the Norwegian Institute of Public Health has reported that the seasonal flu outbreak from December 2019 to March 2020, which corresponds with the 6-month period before the lockdown (pre3), was limited compared to those in previous years [35]. Thus, normal variations due to seasonal flu cannot provide a full explanation for more contact with GPs and somatic hospital services in the 6-month period before lockdown (pre3). The next seasonal flu, expected from December 2020 to March 2021 (post1), did not appear as expected, most likely because of the infection control measures in connection with the COVID-19 outbreak [36, 37]. The increase in the frequency of contact with GPs and somatic hospital services detected in the 6-month period after the lockdown (post1) may be explained by the fact that people had less contact with these services for diseases other than COVID-19 during the first wave of the pandemic [32], and that these consultations accumulated when society started reopening. Furthermore, the increase in contact with GPs and somatic hospital services after the lockdown may be explained by the increased contact between people, which may have caused an increased spread of infections [37].

Finally, the increase in mental health problems during the pandemic [27, 28, 30], may have required additional medical supervision. Studies have reported an increase in depression among older adults during the pandemic, a related increase in the prescription of antidepressant medication [30, 38], and the need for primary health services, such as GPs, and specialist services, such as hospital services [38].

### Strength and limitations

The main strength of the present study is its large population-based survey sample merged with unique national registry data on primary and specialist health care services. This provided objective data regarding the participants’ service use. Despite the large study sample, all the participants were from the middle region of Norway, which may differ from the population in other parts of the country and outside Norway. Furthermore, the study sample was a homogenous group of participants mainly born in Norway, and the results cannot be generalised to other ethnic groups. Although the diagnostic process for dementia was thorough, the diagnosis was based on collected research data without access to imaging or biomarker data which may have caused misclassification. As our goal was to estimate the actual change in service use based on dementia status among younger and older adults, the analysis does not include health-related covariates such as comorbidity and functional level. Finally, the information on dementia status was collected from 2017 to 2019 and may have changed during the study period from September 2018 to September 2021.

## Conclusion

The use of primary care and specialist health services was immediately reduced during the COVID-19 lockdown period. Within primary care services, people with dementia experienced a more pronounced reduction than that experienced by people without dementia; however, age and dementia status only demonstrated small variations. One year after the lockdown, service provisions returned to a level similar to or higher than that of one year before the lockdown for all groups. Our findings indicate that infection control and management limited the scope of action within care services and specialist health services during the lockdown, leaving GPs on the front line to manage medical problems and psychological stress in the population. In any future pandemic, the reallocation of resources for primary health services could make us better equipped to meet the needs of the population.

## Data Availability

The data that support the findings of this study are available from the HUNT database and the Norwegian registry database, Helsedata, but restrictions apply to the availability of these data, which were used under license for the current study, and so are not publicly available. Data are however available from the authors upon reasonable request and with permission of the HUNT database and the Norwegian registry database Helsedata.
